# Synthesis of anti-allergic drugs

**DOI:** 10.1039/c9ra10659f

**Published:** 2020-02-04

**Authors:** Shiyang Zhou, Gangliang Huang

**Affiliations:** Chongqing Key Laboratory of Green Synthesis and Application, Active Carbohydrate Research Institute, Chongqing Normal University Chongqing 401331 China; Key Laboratory of Tropical Medicinal Resource Chemistry of Ministry of Education, College of Chemistry and Chemical Engineering, Hainan Normal University Haikou 571158 China huangdoctor226@163.com

## Abstract

Histamine is formed by the decarboxylation of histidine catalyzed by enzymes. It is an endogenous biologically active substance involved in multiple complex physiological processes as an important chemical transmitter. Histamine receptors have four subtypes, H_1_, H_2_, H_3_ and H_4_, all of which are G protein coupling receptors (GPCRs) with different physiological functions. Histamine plays an important role in the pathophysiological mechanism of allergic diseases, and the antagonistic effect of histamine has become an important way to study anti-allergic drugs, wherein the anti-allergic drugs used in clinical practice are mainly H_1_ receptor antagonists. Currently, there are many varieties of H_1_ receptor antagonists in clinical applications, which can be divided into ethylenediamine antagonists, amino ether antagonists, propylamine antagonists, tricyclic antagonists, piperazine antagonists and piperidine antagonists depending on their chemical structures. This article mainly reviews the research progress of allergic reactions with histamine H_1_ receptor antagonists and expounds the important aspects of the design and synthesis of various new compounds.

## Abstract 2

Histamine is formed by the decarboxylation of histidine catalyzed by enzymes. It is an endogenous biologically active substance involved in multiple complex physiological processes as an important chemical transmitter.

## Graphical abstract

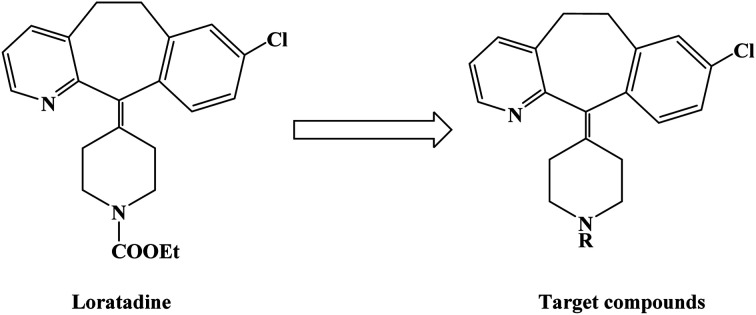

## 1. Introduction

Histamine, chemically known as 4(5)-(2-aminoethyl)imidazole, is an endogenous biologically active substance involved in multiple complex physiological processes as an important chemical transmitter. Histamine is formed by the decarboxylation of histidine catalyzed by enzymes.^1–6^ It is widely found in a variety of plants, animals and microorganisms in nature.^7–9^ In animals, histamines are found in peripheral tissues and in peripheral and central nerves. Histamine is released under endogenous and exogenous stimulations and interacts with receptors. Histamine receptors have four subtypes, H_1_, H_2_, H_3_ and H_4_, all of which are G protein coupling receptors (GPCRs) with different physiological functions.^10–13^

H_1_ receptors are distributed in endothelial cells, smooth muscle, adrenal medulla, heart and central nervous system. Exogenous antigens can cause anaphylaxis, combine with antibody immunoglobulin on target cell mast cell nucleus and granulocyte, change the function of target cell membrane, release histamine and other allergic media, and make histamine distribute in H_1_ receptor function of tissues and organs.^14^ After the activation of the H_1_ receptor, phosphatidase C is activated through G proteins, which promotes the increase in Ca^2+^ concentration, thereby increasing the vasodilation and capillary permeability, leading to plasma exudation, local tissue redness and swelling, and allergic symptoms of bronchial and gastrointestinal smooth muscle contraction.^15–17^ H_2_ receptors are distributed in gastric wall cells, vascular smooth muscles, heart and central nervous system. H_2_ receptor stimulation could promote gastric acid secretion, stimulate the heart and inhibit uterine contraction.^18^ Histamine, acetylcholine and gastrin receptors could be found on the surface of gastric mucosa cells. When the corresponding ligands interact with these receptors, they could activate the secretion of gastric acid. The H_3_ receptor is located in the central and peripheral nerve endings of the presynaptic membrane, and it could function as their receptor, inhibiting histamine release and synthesis.^19–23^ H_3_, as a different receptor, controls the release of other neurotransmitters. H_3_ receptor has important physiological functions, such as cardiac function, gastric acid secretion, anaphylaxis, sleep wake cycle control, cognition and memory, convulsion, diarrhea or reducing gastric acid secretion.^24–30^ The H_4_ receptor is only found in the small intestine, spleen, thyroid and immunocompetent cells. The H_4_ receptor is related to the regulation of immune functions, such as allergic reactions, asthma, cancer and other immune diseases.^31–35^

Anaphylaxis is an allergic disease that occurs when the body is exposed to specific allergens.^36–39^ It could be caused by a variety of exogenous substances like allergens that include heterologous serum (such as tetanus antitoxin), certain animal proteins (such as that of fish, shrimp, and crabs), bacteria, viruses, parasites, animal fur, plant pollen, dust mites in the air, and chemicals and drugs. Allergens could stimulate human B cells to produce immunoglobulin E, which combines with antibodies on human mast cells and sensitized cells, damages the cell membrane and leads to degranulation, and releases histamine, 5-hydroxytryptamine, leukotrienes, bradykinin and other active substances in the granules.^40–46^ The allergic reaction can manifest as skin redness and swelling, rash, itching, patches as well as allergic rhinitis, bronchial asthma, laryngeal edema and other symptoms. The clinical manifestations of anaphylaxis include decreased blood pressure, increased heart rate, pale skin, edema, and shock and even death in severe cases.^47–51^

Histamine plays an important role in the pathophysiological mechanism of allergic diseases, and the antagonistic effect of histamine has become an important way to study anti-allergic drugs.^52–55^ Currently, the anti-allergic drugs used in clinical practice are mainly H_1_ receptor antagonists.^56–60^ H_1_ receptor antagonists were first reported in 1933 to relieve bronchospasm in animals induced by the inhalation of excessive histamine. Since the 1930s, scientists around the world have conducted a large number of structural modifications by using structure activity relationship (SAR) studies, and dozens of classic anti-allergy drugs have been listed for clinical use. In the history of drug development, they have been listed as the first generation H_1_ receptor antagonists. The second generation H_1_ receptor antagonists were marketed after the 1980s. These compounds have characteristics of high selectivity towards the H_1_ receptor, no sedation effect, separation of the antihistamine effect from the central nervous system and fewer side effects. They were known as non-sedation H_1_ receptor antagonists.^61^ The third generation H_1_ receptor antagonists are active optical isomers of the second generation H_1_ receptor antagonists or their metabolites. This generation has greater safety and lower toxic and side effects; thus, their application scope has been expanding in recent years.^62–65^ Currently, there are many varieties of H_1_ receptor antagonists in clinical application, which can be divided into ethylenediamine antagonists, amino ether antagonists, propylamine antagonists, tricyclic antagonists, piperazine antagonists and piperidine antagonists depending on their chemical structures.^66–70^ This article mainly reviews the research progress of allergic reactions with histamine H_1_ receptor antagonists (2011–2017), including the research on the design and synthesis of various new compounds.

## 2. Synthesis

**Fig. 1:**
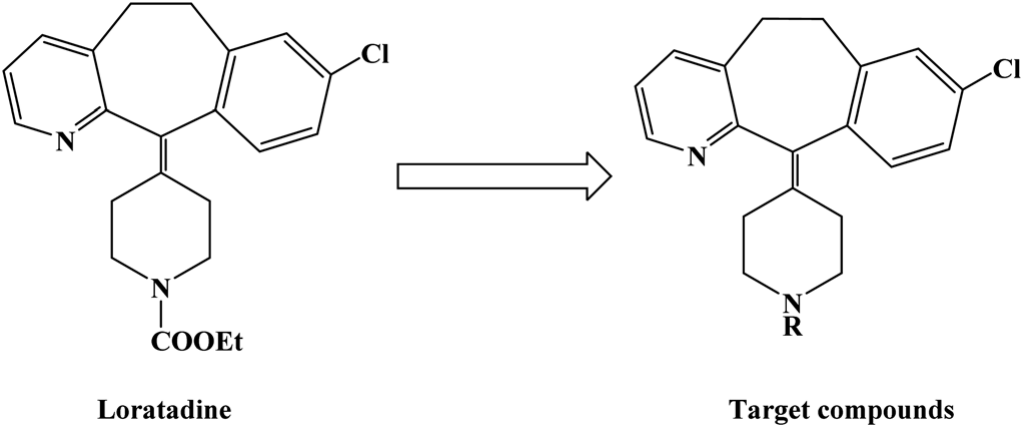
The structural modification of loratadine analogues 4a–h.

**Scheme 1:**
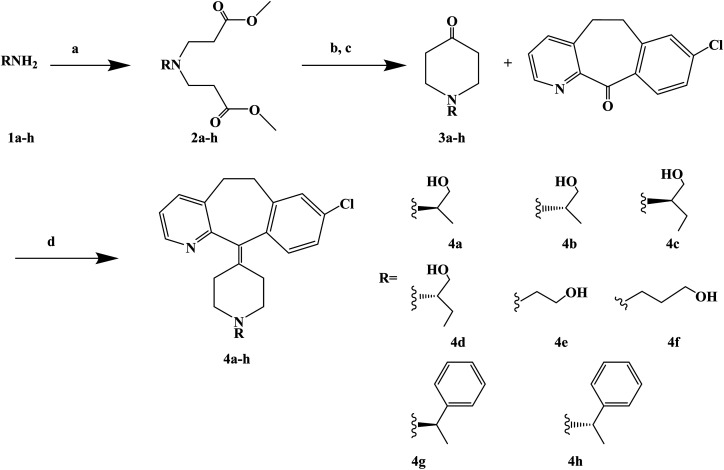
General synthesis of loratadine analogues 4a–h. Reagents and conditions: (a) methyl acrylate, MeOH, 40 °C, 12 h; (b) MeONa/MeOH 30% aq., toluene, 110 °C, 5 h; (c) HCl, 100 °C, 5 h; (d) TiCl_4_/Zn, THF dry, 70 °C, 17 h.

Loratadine is a second-generation antihistamine because it has fewer central nervous system and anticholinergic side effects. Loratadine has a strong therapeutic effect because of its semi-rigid conformation. Lewis *et al.* have reported a series of compounds based on loratadine that have both H_1_ receptor antagonist activity and 5-lipoxygenase inhibitory activity. Liu *et al.* successfully synthesized a series of loratadine derivatives with C-alkyl butenamide, and found that several of them were able to inhibit histamine induction. Yue W. *et al.*^71^ further optimized the structure of loratadine based on previous studies, and designed and synthesized a series of loratadine derivatives (Fig. 1). In structural optimization, hydroxyl groups and chiral centers were introduced into the molecules to enhance their affinity for H_1_ receptors. In the synthetic process (Scheme 1), a series of chiral hydroxyl groups containing *N*-substituted-4-piperidone were obtained by Michael addition. Subsequently, a Dieckmann cyclization and decarboxylation were performed with chiral or non-chiral aminoalcohols and methyl acrylate as raw materials, affording a yield of 35%. A variety of carbonyl groups were obtained by coupling piperidone with a tricyclic ketone using a low-priced titanium catalyst to obtain a series of target compounds at a yield of approximately 25%. The synthetic route is characterized by mild reaction conditions, good versatility and simple operation.

**Fig. 2:**
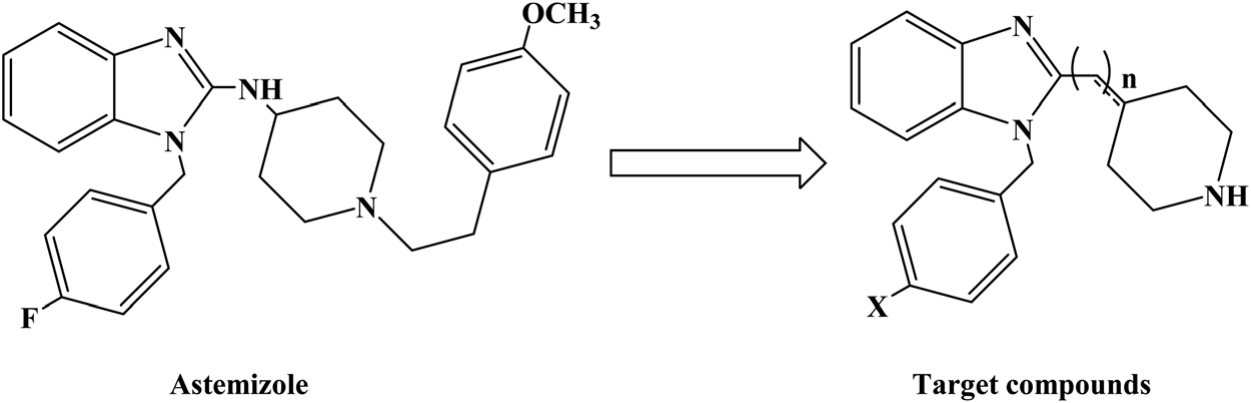
The structural modification of benzimidazole derivatives 12a–f.

**Scheme 2:**
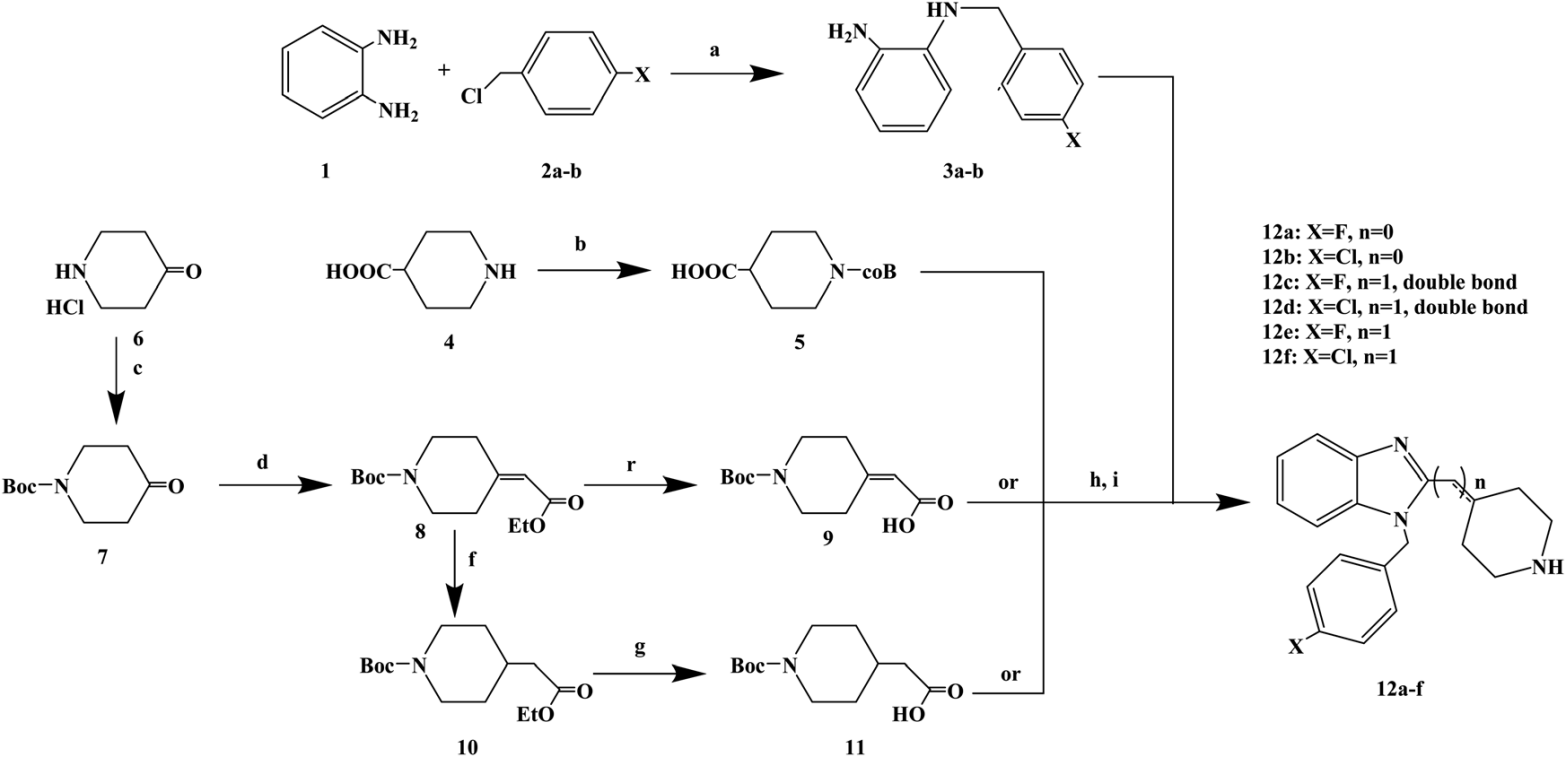
General synthesis of benzimidazole derivatives 12a–f. Reagents and conditions: (a) K_2_CO_3_, DMF, 50 °C, 6 h; (b) Boc_2_O, 1 mol L^−1^ NaOH, THF, 25 °C, 2 h; (c) Boc_2_O, 2 mol L^−1^ NaHCO_3_, THF, 25 °C, 6 h; (d) ethyl diethoxyphosphoryl acetate, K_2_CO_3_, DMF, 78 °C, 12 h; (e) 1 mol L^−1^ NaOH, CH_3_OH, 25 °C, 30 min; (f) 10% Pd/C, H_2_, C_2_H_5_OH, 25 °C, 7 h; (g) 1 mol L^−1^ NaOH, CH_3_OH, 25 °C, 30 min; (h) ethyl chloroformate, Et_3_N, CH_2_Cl_2_, 0 °C, 30 min; (i) AcOH, reflux, 2 h.

Astemizole (Fig. 2) is an effective histamine H_1_ receptor antagonist (*K*_i_ = 2 nmol L^−1^). However, it could lead to arrhythmias with acquired long QT syndrome, which includes torsades DE pointes, and sudden death due to the blockage of rapidly activated delayed rectification K^+^ current *I*_Kr_ and its underlying human ether-a-gogo related gene (hERG) K^+^ channel (IC_50_ = 0.9 nmol L^−1^). In order to find a new type of H_1_ receptor antagonist with low hERG blocking activity, Xiao J. *et al.*^72^ carried out systematic structural modification based on astemizole (Fig. 2). The synthetic route of the benzimidazole derivative 12a–12f was summarized in Scheme 2. In addition, compounds 9 and 11 were synthesized from compound 6 according to previous literature. The compounds 12a–12f were produced by following acylation, cyclation, and deprotection procedures. The synthetic route was characterized by mild reaction conditions, high yield and good versatility.

**Scheme 3:**
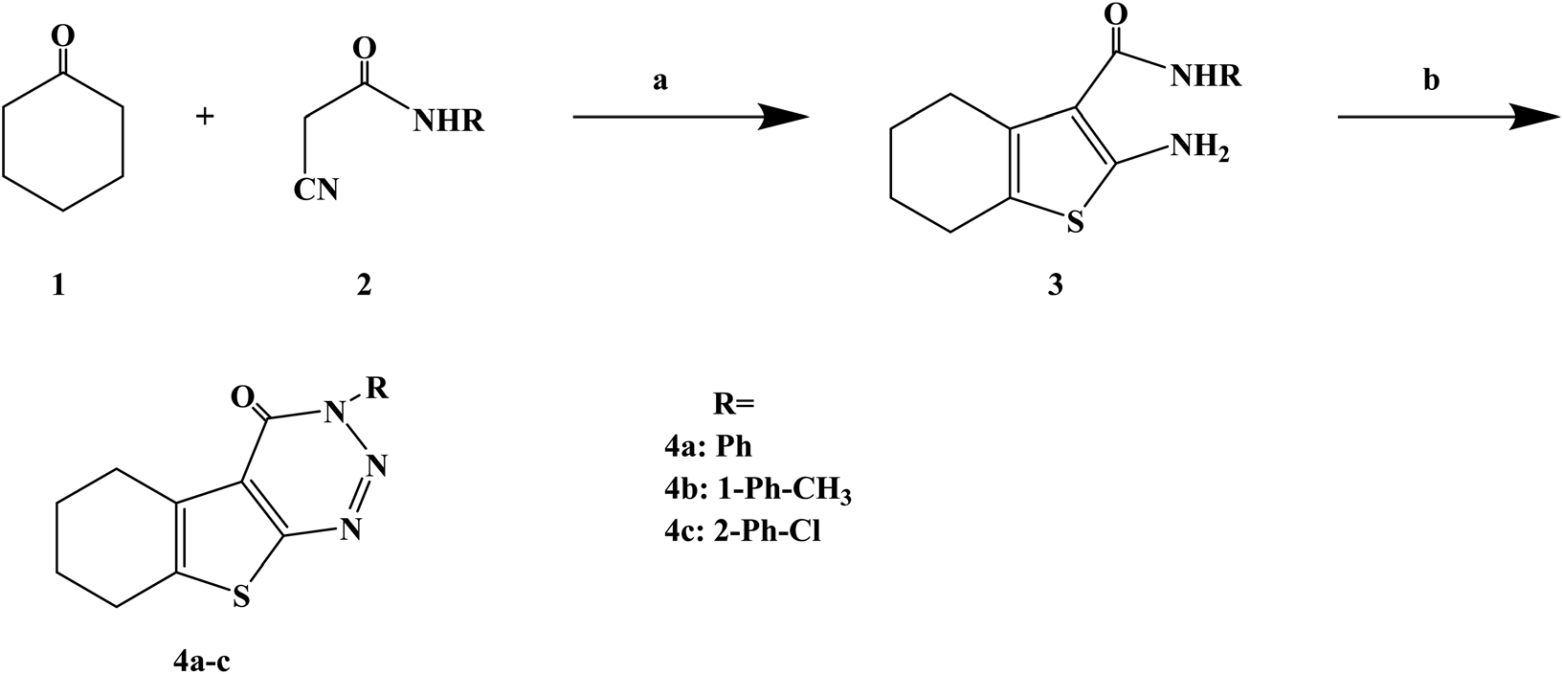
General synthesis of 3*H*-benzo[4,5]thieno[2,3-*d*][1,2,3]triazin-4-ones 4a–c. Reagents and conditions: (a) CH_3_COOH/CH_3_COONH_4_, glacial acetic/cyclohexane, reflux 10 h; (b) NaNO_2_/HCl, glacial acetic, 0–5 °C.

Numerous uses of substituted triazines have been reported, such as anti-inflammatory, analgesic activity, purine confrontation, antitumoral and trypanocidal activity. Very little studies have been conducted on heterocyclic compounds such as 1, 2, 3-triazine and other triazines, this kind of compounds and similarity based on the structure of these compounds and other triazines like 1,2,4-triazine and 1,3,5-triazin. It was found that 1,2,3-triazine derivatives possess prostaglandin inhibition property, analgesic, anti-inflammatory and antihistaminic properties. Gollapalle L. *et al.*^73^ further optimized 1,2,3-triazine on the basis of previous studies, and designed and synthesized three new compounds with antihistamine effects. In the process of synthesis (Scheme 3), the starting compounds 2-amino-3-(*N*-substituted carboxamido)-4 and 5-tetramethylene thiophenes (compounds 3a–c) were synthesized in three steps by adapting the Gewald reaction. Afterwards, compounds 3a–c were diazotized to yield a series of 3-substituted amino-5,6-tetramethylene thieno[2,3-*d*][1,2,3]-triazin-4(3*H*)-ones (compounds 4a–c). In this reaction, the starting compounds 2-amino-3-(*N*-substitutes carboxamido)-4 and 5-tetramethylene thiophenes (compounds 3a–c) react with NaNO_2_ in the presence of HCl yielding the respective triazine-4-ones. The synthetic route was characterized by mild reaction conditions, simple operation and cheap and easy to obtain reagents.

**Fig. 3:**
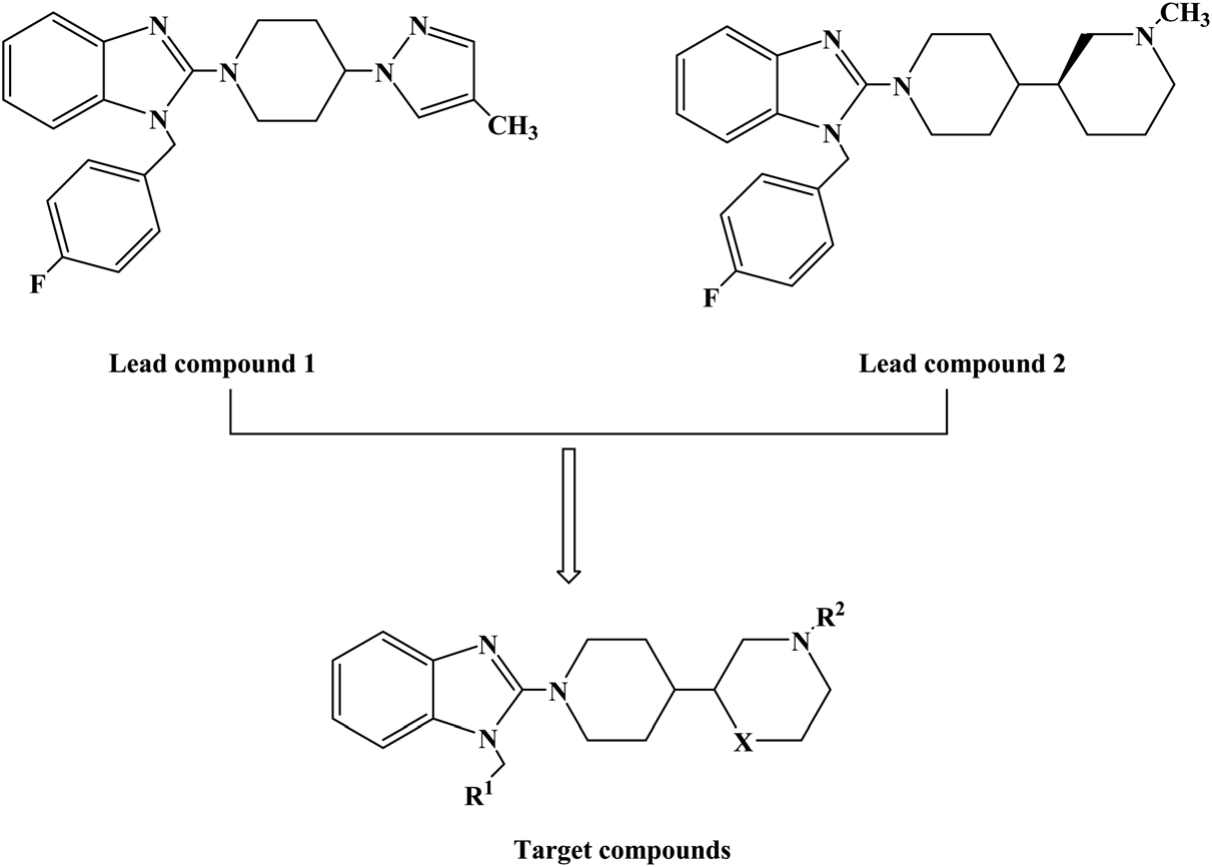
The structural modification of 2-(piperidin-3-yl)-1*H*-benzimidazole analogues 5a–n, 6a–d and 7a–h.

**Scheme 4:**
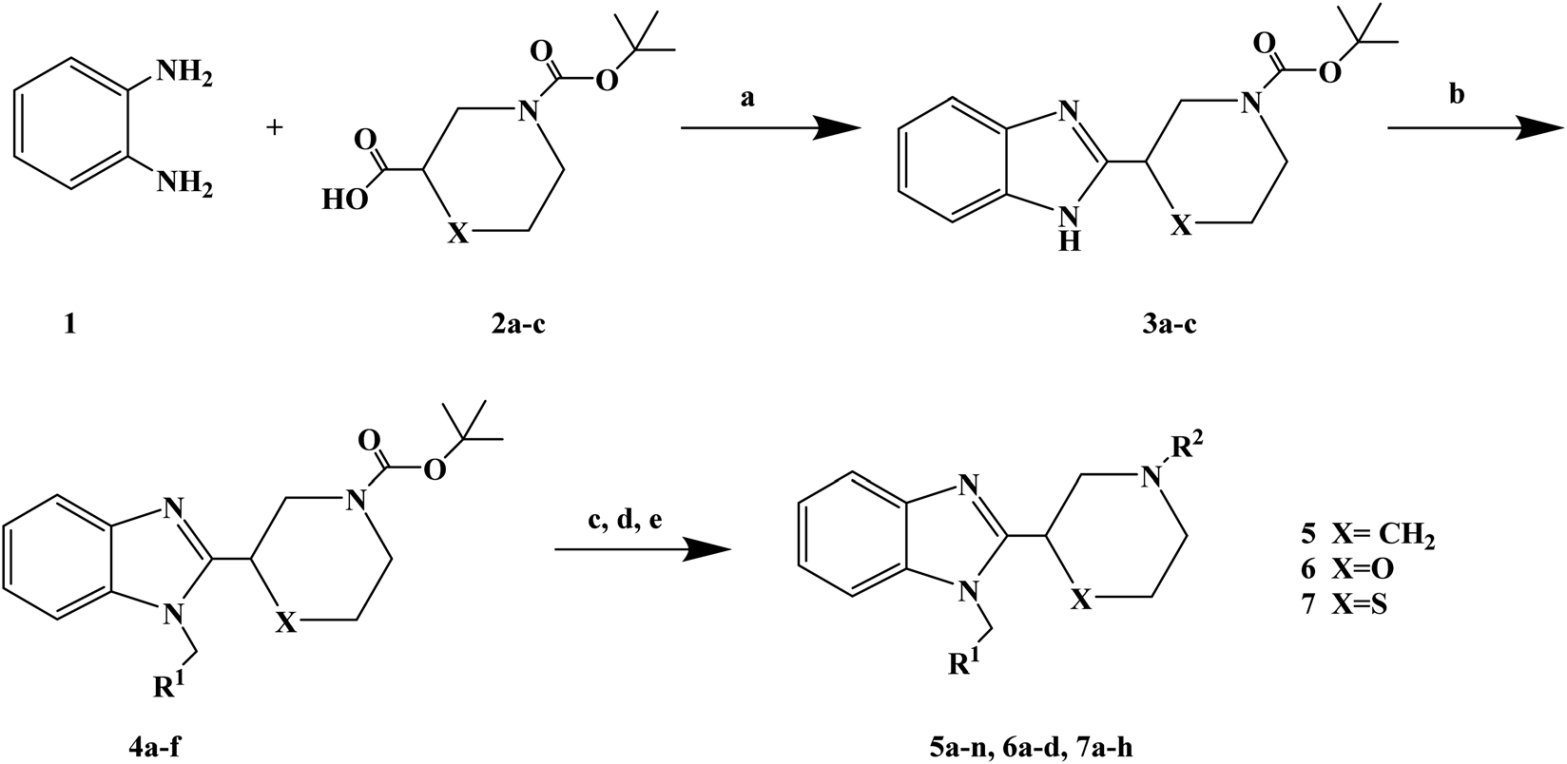
General synthesis of 2-(piperidin-3-yl)-1*H*-benzimidazole analogues 5a–n, 6a–d and 7a–h. Reagents and conditions: (a) EtOH, 120 °C; (b) R^1^CH_2_Br, K_2_CO_3_, DMF, 80 °C; (c) TFA, CH_2_Cl_2_; (d) R^2^–(=O)H, Na(OAc)_3_BH or R_2_X, Et_3_N, THF; (e) separation of enantiomers by chiral SFC when chiral acids were not used.

1-(4-Fluorobenzyl)-2-(4-(4-methyl-1*H*-pyrazol-1-yl)piperidin-1-yl)-1*H*-benzo[*d*]imidazole (lead compound 1, Fig. 3) is a high affinity and selective H_1_-antihistamine. The lead compound 1 exhibits limitations of poor metabolic stability and solubility. Further optimization of compound 1 led to the development of (*R*)-1-(4-fluoro benzyl)-2-(1-methylpiperidine-3-acyl)-1*H*-benzimidazole (lead compound 2, Fig. 3) with high affinity for H_1_-antihistamine. The lead compound 2 showed improved selectivity and stability for hERG channel when compared with lead compound 1. Satheesh B. *et al.*^74^ performed relevant structure activity relationship (SAR) studies, determining that a change in the direction of the basic center in lead compound 2 was a key step in establishing the required H_1_ affinity and selectivity. Based on this discovery, Satheesh B. *et al.* designed and synthesized a series of H_1_-antihistamines (Fig. 3) after structural optimization of the lead compounds 1 and 2. In this process, different substituents R^1^ were introduced into the imidazole ring. The polar group R^2^ was added to the piperidine ring nitrogen, hence, p*K*_a_ and/or log *P* were reduced by the electron attraction effect. The synthesis of analogues described in Scheme 4 relies on the formation of the benzimidazole core (compound 3), after the coupling of a suitably protected amino acid (compound 1) with *O*-phenylene diamine (compound 2). Introduction of the appropriate arene-containing moiety R^1^ was accomplished by alkylation of compound 3. The final step included the alkylation or reductive amination and the removal of Boc to obtain the final product (compounds 5a–n, 6a–d and 7a–h). Further separation of enantiomers was achieved by chiral SFC when chiral acids were not used for the synthesis of benzimidazole. The synthetic route was characterized by mild reaction conditions, cheap and easy to obtain reagents, relatively high yield and good versatility.

**Scheme 5:**
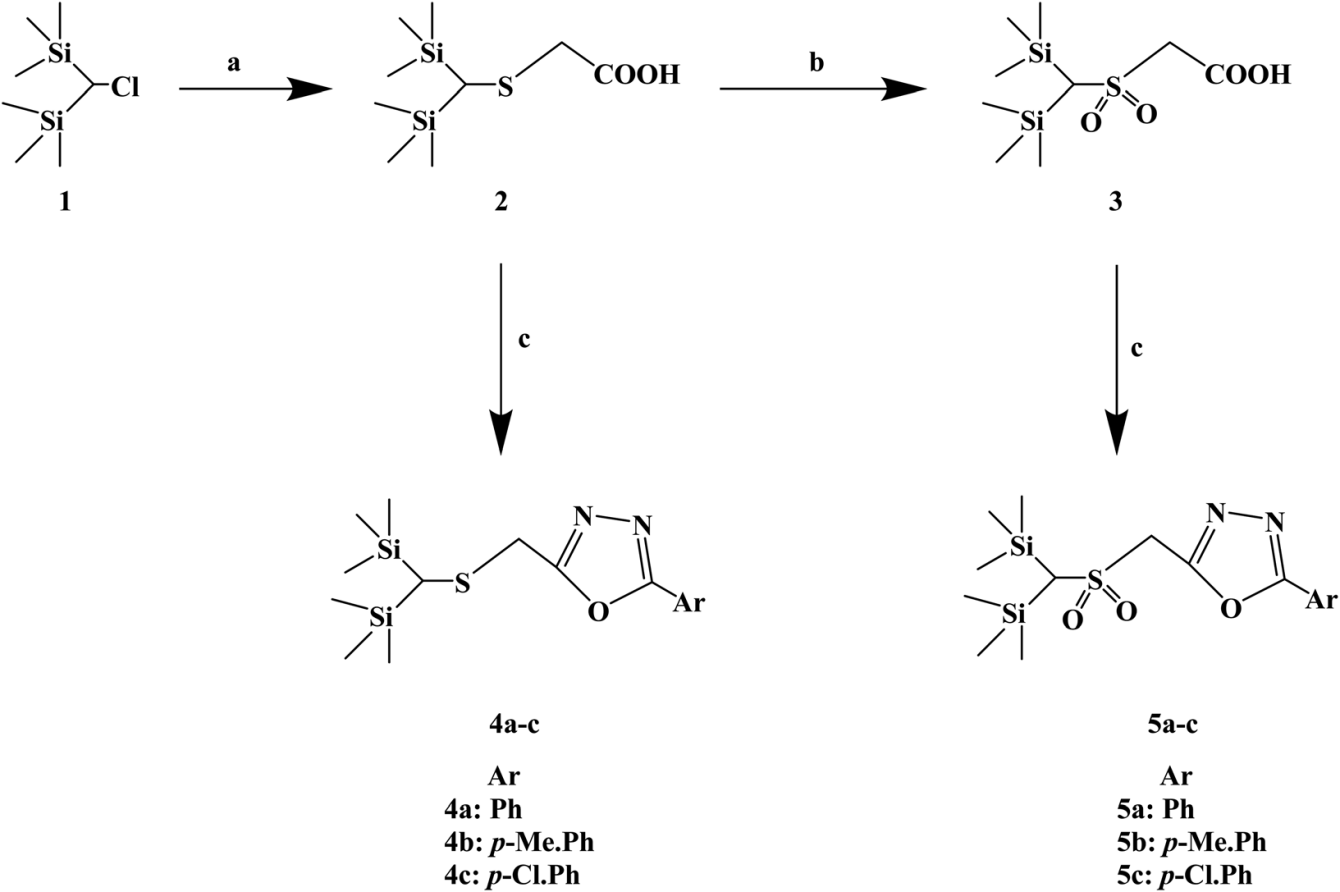
General synthesis of 2-((bis(trimethylsilyl) methylthio/methylsulfonyl) methyl)-5-aryl-1,3,4-oxadiazoles. Reagents and conditions: (a) HSCH_2_COOH, MeOH, NaOH, reflux 4 h; (b) H_2_O_2_, AcOH, 24 h; (c) ArCONHNH_2_, POCl_3_, reflux 6 h.

Silicon substitution (C/Si exchange) has attracted extensive attention in drug-like scaffold materials due to its unique biological activity, chemical reactivity and physical and chemical properties compared with the parent carbon compounds. 1,3,4-Oxadiazole derivatives have been reported as promising antiallergic drugs. Dinneswara R. *et al.*^75^ designed and synthesized a variety of substituted 1,3,4-oxadiazole compounds (Scheme 5) in a study related to silicon substitution drugs, and applied them to the study of antiallergic drugs. In the structural design process, –Ph, 4-Me–Ph and 4-Cl–Ph substituents were selected. In the process of synthesis, 2-(bis(trimethylsilyl)methylthio) acetic acid (compound 2) was synthesized by the reaction of bis(trimethylsilyl)chloromethane (compound 1) and mercaptoacetic acid in the presence of NaOH/MeOH. Compound 3 was subsequently oxidized in the presence of H_2_O_2_/CH_3_COOH. The required heterocyclic compounds, 2-((bis(trimethylsilyl)methylthio)methyl)-5-aryl-1,3,4-oxadiazoles (compound 4) and 2-((bis(trimethylsilyl)-methylsulfonyl) methyl)-5-aryl-1,3,4-oxadiazoles (compound 5) were synthesized by the reaction of compound 2/compound 3 with benzohydrazides in the presence of phosphorus oxychloride. The synthetic route has high reaction yields (79% to 88%), mild reaction conditions and conventional reagents.

**Scheme 6:**
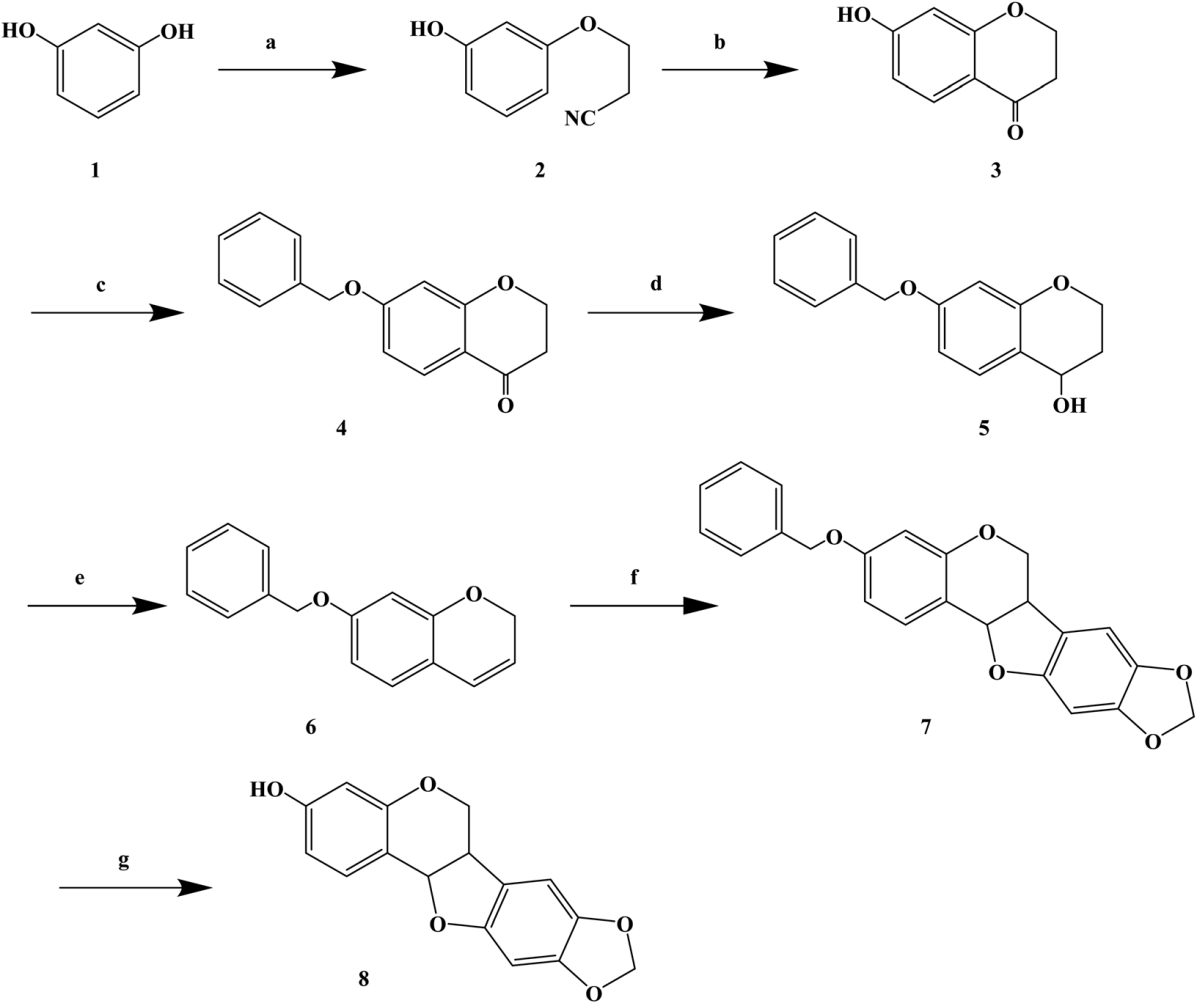
General synthesis of (±)-maackiain. Reagents and conditions: (a) acrylonitrile, triton B, NaOH, 100 °C, 10 h; (b) H_2_SO_4_, CH_3_COOH, H_2_O, 100 °C, 8 h; (c) benzyl chloride, NaI, K_2_CO_3_, acetone, 60 °C, 18 h; (d) NaBH_4_, ethanol, rt, 3 h; (e) *p*-toluenesulfonic acid, toluene, 110 °C, 2 h; (f) PCl_2_(PhCN)_2_, KoAc, DMF, 40 °C, 4 days; (f) H_2_–Pd/C, 3 h.

Over the years, Kujin has been widely used to treat allergic diseases and many others. Phytochemical studies have shown that it contains quinolizidine, alkaloids, flavonoids, and triterpenoids. Kujin and its active components have many biological properties, such as anti-inflammatory, antiasthmatic, antitumor and antibacterial. Hiroyuki M. *et al.*^76^ found that the extract of Kujin has inhibited the up-regulation of H1R and IL-4 gene expression in rats sensitized by TDI, and confirmed (*R*)-maackiain as the main anti-allergy component of Kujin. (*R*)-Maackiain is found in low concentration in plants, which made it difficult to extract; so, it was obtained by chemical synthesis (Scheme 6). In the process of synthesis, maackiain bis(benzonitrile) palladium(ii) dichloride [PdCl_2_(PhCN)_2_] was prepared as reported previously. The synthesis of (*R*)-3-benzylmaackiain was performed as follows. To a solution of 7-benzyloxy-2*H*-1-benzopyran and 2-bromo-4,5-methylene dioxyphenyl in anhydrous DMF suspended in potassium acetate, PdCl_2_(PhCN)_2_ was added in one portion. The resulting suspension was stirred at 40 °C. After 24 h, the addition of PdCl_2_(PhCN)_2_ to the resulting reaction mixture was carried out. The latest procedure was repeated after 48 h and 72 h. Finally, the resulting mixture was stirred for additional 24 h. This synthetic route achieved the first chemical synthesis of (*R*)-maackiain, but it had disadvantages, such as long synthetic route, low yield, the use of precious metal Pd in the synthesis process, and long overall reaction time, which need to be further optimized.

**Scheme 7:**
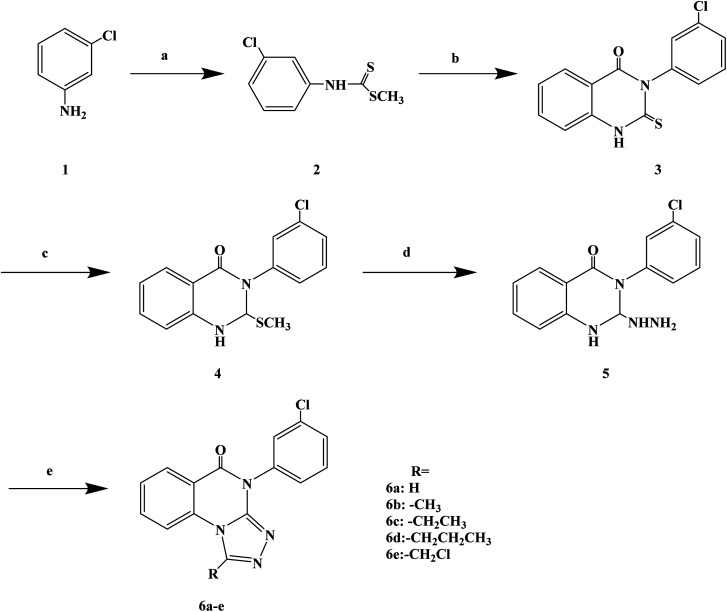
General synthesis of 1-substituted-4-(3-chlorophenyl)-[1,2,4]triazolo[4,3-*a*] quinazolin-5(4*H*)-ones 6a–e. Reagents and conditions: (a) CS_2_, NaOH, stirring 30 min, (CH_3_)_2_SO_4_; (b) methyl anthranilate/EtOH, reflux 19 h; (c) NaOH/EtOH, string 2 h; (d) NH_2_NH_2_, H_2_O/EtOH, reflux 24 h; (e) reflux 29 h.

Quinazolinones have a wide range of biological and pharmacological effects, such as analgesic, anti-inflammatory, antifungal, antiviral and antihistamine activities. Veerachamy A. *et al.* proved that quinazolinone derivatives have strong antihistamine activity with alow sedative effect. Manavalan G. *et al.*^77^ designed and synthesized a series of 1-substituted-4-(3-chlorophenyl)-[1,2,4]triazolo[4,3-*a*]quinazolin-5(4*H*)-ones (Scheme 7) based on the structural modifications of quinazolone. In that process, the triazolo group has been substituted with different groups (–H, –CH_3_, –CH_2_CH_3_, –(CH_2_)_2_CH_3_, –CH_2_Cl) to obtain compounds with improved biological activity. In the process of synthesis, compound 3 (3-(3-chlorophenyl)-2-thioxo-quinazolin-4(3*H*)-one) was belonged to the key intermediate. Dithiocarbamic acid methyl ester was prepared with 3-chloro aniline, carbon disulfide and sodium hydroxide in dimethyl sulfoxide, then methylated with dimethyl sulfate, which was reacted to produce the required compound 4 in ethanol has a good yield (86%). Compound 3 was dissolved in a solution of 2% ethanol sodium hydroxide, stirred at room temperature, and methylated with dimethyl sulfate to give 3-(3-chlorophenyl)-2-(methylthio) quinazolin-4(3*H*)-one (compound 4). The nucleophilic displacement of the methylthio group of compound 4 with hydrazine hydrate was carried out using ethanol as the solvent, affording compound 5 (3-(3-chlorophenyl)2-hydrazinyl-quinazolin-4(3*H*)-one). Finally, the compound 5 with a variety of one carbon donors such as acetic acid, propionic acid, formic acid, butyric acid and chloroacetyl chloride under refluxing provided 1-substituted-4-(3-chlorophenyl)-[1,2,4]triazolo[4,3-*a*] quinazolin-5(4*H*)-ones (compounds 6a–e). The synthetic route has high reaction yield (80% to 85%), mild reaction conditions and uses conventional reagents.

**Scheme 8:**
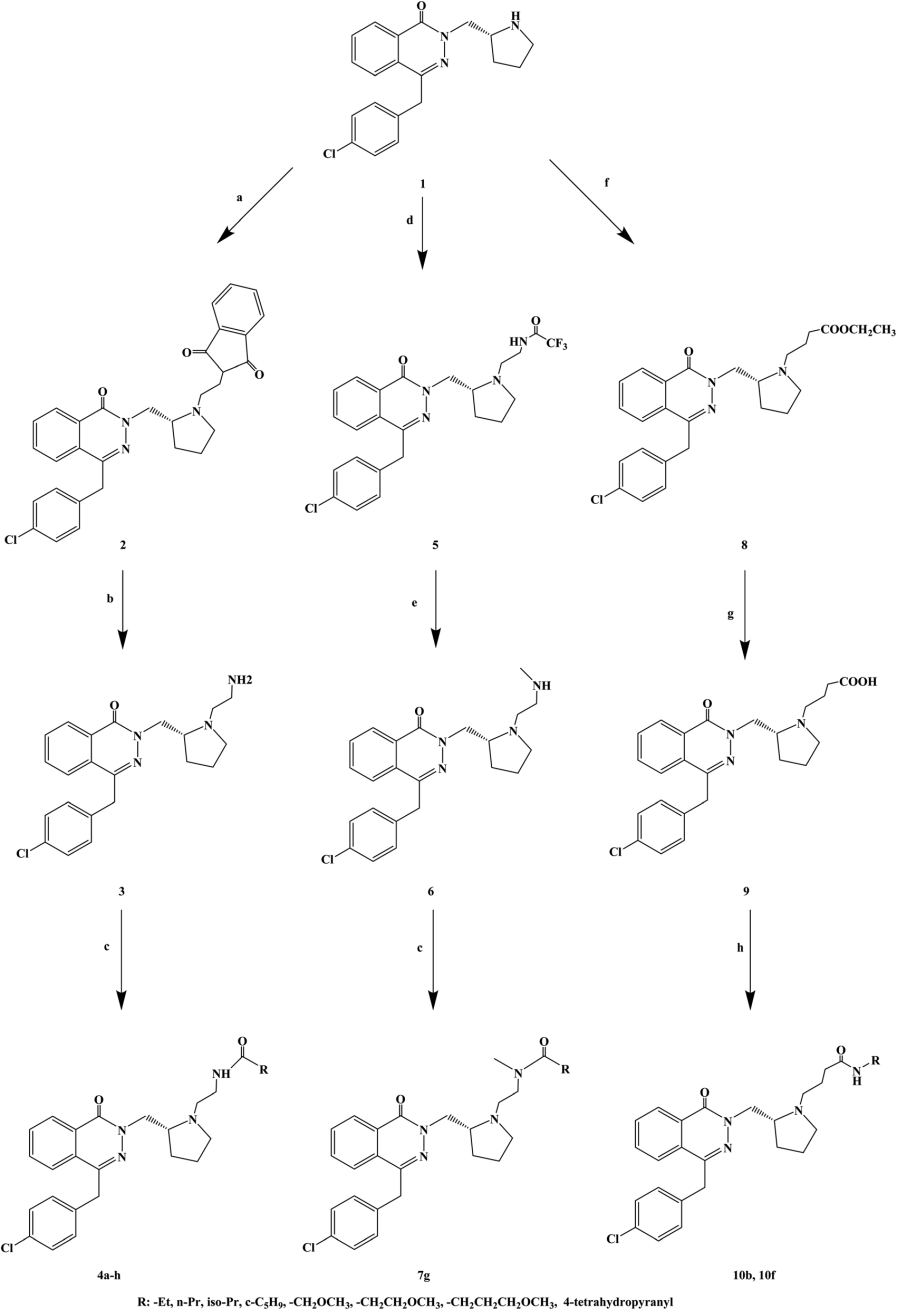
General synthesis of phthalazinone amide 3a–g, 7a–g and 10a–h. Reagents and conditions: (a) *N*-(2-bromoethyl)phthalimide, K_2_CO_3_, 2-butanone, 80 °C, 18 h; (b) NH_2_NH_2_·H_2_O, EtOH, 80 °C, 1.25 h; (c) RCO_2_H, TBTU, Et_3_N, DMF, 2 h, or RCOCl, Et_3_N, DCM; (d) 2,2,2-trifluoro-*N*-(2-iodoethyl)acetamide, DIPEA, 2-butanone; (e) (i) MeI, K_2_CO_3_, DMF; (ii) K_2_CO_3_, H_2_O, MeOH; (f) BrCH_2_CH_2_CH_2_CO_2_Et, K_2_CO_3_, DMF, 150 °C, microwave, 110 min; (g) NaOH, H_2_O, MeOH, 20 °C, 1 h; (h) TBTU, Et_3_N, DMF.

The compounds containing the phthalazinone scaffold were obtained through structural optimization and subsequently used as histamine H_1_ receptor antagonists for the treatment of allergic reactions. These compounds bind to both H_1_ and H_3_ receptors. Based on the structure optimization of phthalazinone, Panayiotis A. *et al.*^78^ introduced different substituents, and designed and synthesized a series of novel phthalazinone amide compounds (Scheme 8). In the molecular structure design, -Et, *n*-Pr, iso-Pr, c-C_5_H_9_, –CH_2_OCH_3_, –CH_2_CH_2_OCH_3_, –CH_2_CH_2_CH_2_OCH_3_, 4-tetrahydropyranyl were selected as the substituent. During the synthetic procedure, compound 1 was alkylated with *N*-(2-bromoethyl) phthalimide to give compound 2 (yield 87%), and then, deprotected using hydrazine to afford compound 3 with 77% yield. Afterwards, compound 3 was acylated with a variety of carboxylic acids in the presence of TBTU to give compounds 4d, 4f, 4g and 4h, or with the corresponding acid chloride to give compounds 4a, 4b, 4c and 4e. Alkylation of compound 1 with 2,2,2-trifluoro-*N*-(2-iodoethyl)acetamide resulted in trifluoroacetamide (compound 5), which was alkylated with iodomethane and deprotected, to obtain the secondary amine (compound 6). The latter was acylated in the presence of 4-methoxybutanoic acid and TBTU to obtain tertiary amide (compound 7g). Compound 1 reacted with ethyl 4-bromobutyrate to give compound 8, which was converted to a carboxylic acid 9, and finally, transformed into amides 10b and 10f. The synthetic route has good versatility and uses conventional reagents, but the partial yield was low (yield 29%), which should be further improved.

**Fig. 4:**
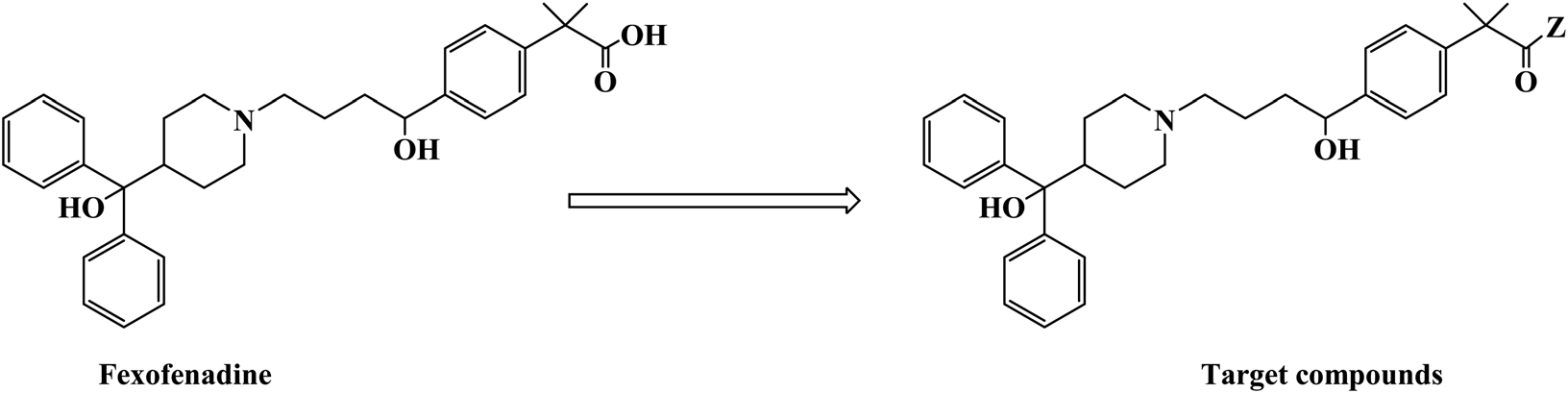
The structural modification of fexofenadine derivatives 3a–e.

**Scheme 9:**
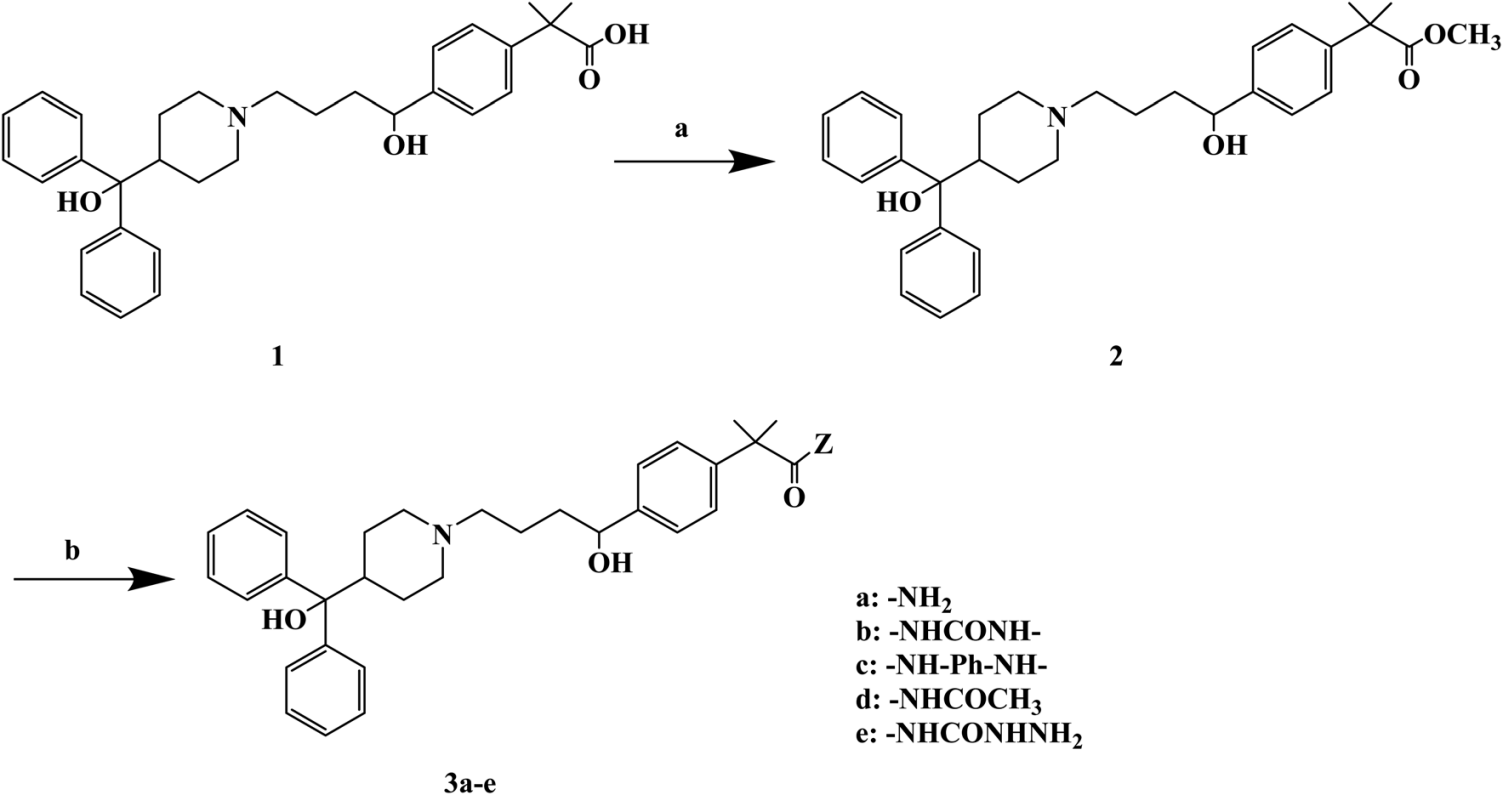
General synthesis of fexofenadine derivatives 3a–e. Reagents and conditions: (a) MeOH, H_2_SO_4_/H_2_O, reflux 7–8 h; (b) amine, reflux 2–3 h.

H_1_ antagonists are competitive drugs that inhibit histamine action on tissues containing H_1_. Fexofenadine (2-[4-[1-hydroxy-4-[4-(hydroxydiphenylmethyl)piperidino]butyl]phenyl]-2-methylpropanoic acid) (Fig. 4) is a selective and peripherally acting H_1_ receptor antagonist and is a nonsedative active metabolite of terfenadine. Fexofenadine was clinically used to treat seasonal allergic rhinitis, but there were associated adverse effects such as headache, throat irritation, viral infection, nausea, dysmenorrhea, drowsiness, dyspepsia and fatigue. Saeed A *et al.*^79^ used fexofenadine as the lead compound (Fig. 4), and designed and synthesized fexofenadine derivatives with hydrazine hydrochloride, urea, *p*-phenelediamine, acetamide and semicarbazide (Scheme 9). Herein, carboxylbenadine 2-methylpropionamide and benadrylamide derivatives were synthesized using carboxylbenadine as the nucleophilic substituent and tertiary C-19 was replaced by a benzene ring. The carboxyl group on the tertiary C-19 was esterified by a condensation reaction, and the nucleophilic substitution reaction on the acyl group formed a tetrahedral intermediate, which transformed back to the carbonyl group after the leaving group was eliminated. On the basis of the above reaction mechanism, various amines were reacted to produce amide compounds, and various reactions were identified. This synthetic route was characterized by a high yield, short reaction time and good versatility.

## 3. Conclusions

Histamine plays an important role in the pathophysiological mechanism of allergic diseases, and the antagonistic effect of histamine has become an important way to study anti-allergic drugs. The anti-allergic drugs used in clinical practice were mainly H_1_ receptor antagonists. This article mainly reviews the research progress of allergic reactions with histamine H_1_ receptor antagonists and expounds the important aspects of design, synthesis and biological activity of various new compounds. These novel H_1_ receptor antagonists, with regards to their chemical structure design, are mostly obtained by optimizing the structure of the reported compounds with antiallergic activity (including natural products, Schemes 6 and 9) or already sold drugs. These compounds have novel structures, and the design of H_1_ receptor antagonists in the future will tend to optimize the structure of natural products as lead compounds. In the process of synthesis, most synthetic routes use conventional solvent reaction to synthesize target compounds. These synthetic routes were characterized by simple operation, mild reaction conditions, cheap reagents and a good overall yield, which will provide certain foundation for future industrialization. However, some synthetic routes use multiple catalysts or precious metals (such as Pd, Scheme 6), or the reaction time was too long (Scheme 7). In the selection of synthesis methods, researchers should focus on the modern synthesis methods, such as microwave synthesis, and the organic synthesis process will have more room for development. As can be seen from the reagent and reaction condition perspective, the choice direction in the future will be to use cheap and readily available raw materials, conventional solvents, no catalyst or conventional catalyst, and medium or low reaction temperature.

## Conflicts of interest

The authors declare that they have no conflict of interest.

## Supplementary Material
